# Hendra Virus Infection in Dog, Australia, 2013

**DOI:** 10.3201/eid2112.151324

**Published:** 2015-12

**Authors:** Peter D. Kirkland, Melinda Gabor, Ian Poe, Kristie Neale, Kim Chaffey, Deborah S. Finlaison, Xingnian Gu, Paul M. Hick, Andrew J. Read, Therese Wright, Deborah Middleton

**Affiliations:** Elizabeth Macarthur Agriculture Institute, Menangle. New South Wales, Australia (P.D. Kirkland, M. Gabor, D.S. Finlaison, X. Gu, P.M. Hick, A.J. Read);; North Coast Local Lands Services (formerly Mid Coast Livestock Health and Pest Authority), Kempsey, New South Wales, Australia (I. Poe);; Macksville Veterinary Clinic, Macksville, New South Wales, Australia (K. Neale, K. Chaffey);; University of Sydney, Camden, New South Wales, Australia (P.M. Hick);; NSW Department of Primary Industries, Orange, New South Wales, Australia (T. Wright);; Australian Animal Health Laboratory, Geelong, Victoria, Australia (D. Middleton)

**Keywords:** Hendra virus, dog, natural infection, pathology, virology, viruses, Australia

## Abstract

Hendra virus occasionally causes severe disease in horses and humans. In Australia in 2013, infection was detected in a dog that had been in contact with an infected horse. Abnormalities and viral RNA were found in the dog’s kidney, brain, lymph nodes, spleen, and liver. Dogs should be kept away from infected horses.

Hendra virus (HeV) is a paramyxovirus (genus *Henipavirus*) (*1*) that causes respiratory and neurologic disease in horses and humans; the case-fatality rate is >60%. Fruit bats are the reservoir hosts (*2*,*3*) and excrete virus in urine (*4*). Disease outbreaks among horses occur sporadically along the eastern coast of Queensland and New South Wales, Australia. Infection of veterinarians after close contact with infected horses presents a serious occupational hazard. After confirmation of Hendra infection in horses, an affected farm is quarantined by animal health authorities. All horses, cats, and dogs determined to be at risk for infection are monitored for clinical signs and tested for virus until they are no longer considered to be potentially infected. Cats and dogs are included in this surveillance because they have been shown to be susceptible to experimental infection with HeV (*5*; D. Middleton, unpub. data). In July 2013, during investigation of HeV infection in a horse near Macksville, New South Wales, Australia, infection was also detected in a dog on the same farm.

## The Study

The infected horse was a 6-year-old Australian stock horse gelding. HeV RNA was detected by quantitative reverse transcription PCR (qRT-PCR) in EDTA-treated blood (cycle threshold [C_t_] 26.82), serum (C_t_ 30.87), and nasal swab samples (C_t_ 34.56) collected on July 4, 2013. Later that day, the horse was killed by shooting. During follow-up investigations on July 6, negative HeV results (qRT-PCR and ELISA) were obtained from whole blood, serum, and nasal swab samples collected from 2 additional horses; whole blood and oral swab samples collected from 2 dogs; and oral swab samples collected from a third dog. These dogs were from the same farm as the HeV-positive horse.

Twelve days later, additional blood samples were collected (placed in EDTA or allowed to clot) from the 3 dogs, and oral swab samples were collected from 1 of these dogs (a 6-year-old cross-bred female fox terrier). HeV RNA was detected in the EDTA-treated blood (C_t_ 31.48) and serum (C_t_ 34.01), but not from the oral swab samples, from this dog. Results from all samples from the other dogs were negative by qRT-PCR and ELISA. Serum from the dog with positive results by qRT-PCR gave a weak positive result by ELISA and a virus neutralization titer of 8. The dog showed no signs of ill health, although it had winced several times, suggesting discomfort or pain. Because the transmission risk posed by the dog was uncertain, it was euthanized 14 days after collection of the first samples. Blood (placed in EDTA or allowed to clot); oral, nasal, rectal, and vaginal swab samples; and urine were collected immediately thereafter. The cadaver was immediately transported to the laboratory, and a postmortem examination was conducted later that day.

**Figure 1:**
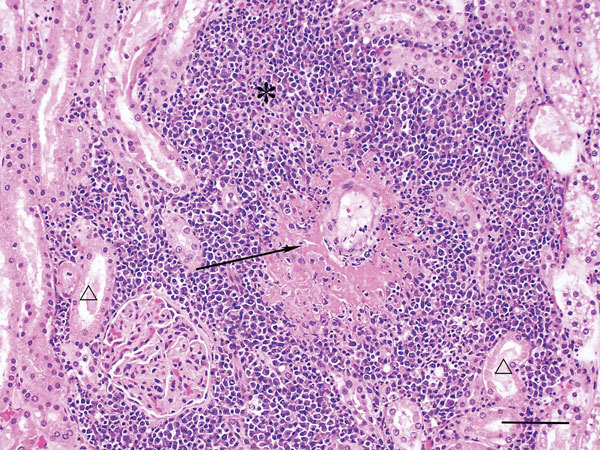
Kidney of dog infected with Hendra virus, showing marked vasculitis (arrow) and inflammatory infiltrates (*) effacing renal tubules (△). Scale bar indicates 75 μm.

**Figure 2:**
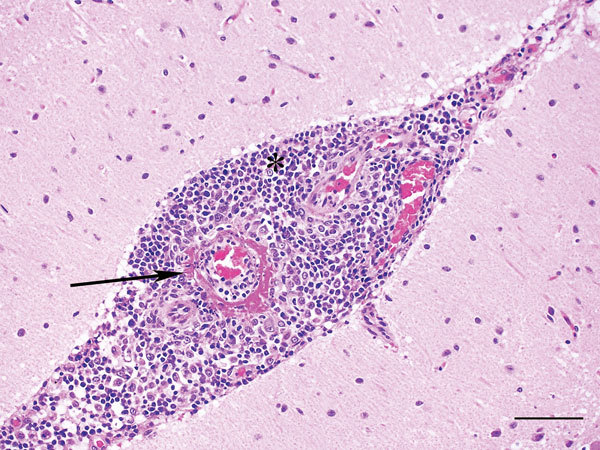
Cerebellum of dog infected with Hendra virus, showing expansion of the meninges with inflammatory infiltrates (*) and marked vasculitis (arrow). Scale bar indicates 75 μm.

No external gross abnormalities were detected. Internal examination revealed diffuse marked reddening of all lung lobes and overlying dark patchy discoloration of dependent lobes; abundant frothy tracheal and bronchial fluid; enlargement and diffuse reddening of bronchial, tracheobronchial, and mandibular lymph nodes; prominent and diffuse reddening of both tonsils; and prominent white streaks at the corticomedullary junction of both kidneys. The spleen and liver were enlarged with rounded edges, and the liver had a mild cobblestone pattern (Table 1). Histopathology findings closely aligned with gross findings; lesions in the brain were also histologically detected. The predominant lesion, found in decreasing severity in kidney, brain, lymph nodes, spleen, liver, intestine, and lung, was fibrinoid necrosis of vessels with marked segmental to diffuse vasculitis, disruption of subendothelial tunica intima, and expansion with thick bands of deeply eosinophilic hyaline to fibrinoid material admixed with karyorrhectic debris and degenerate neutrophils (Figure 1). Surrounding inflammatory infiltrates (plasma cells, lymphocytes, and karyorrhectic debris) often effaced and replaced surrounding normal structures. Cerebral and cerebellar meninges were moderately expanded with lymphocytes, plasma cells, and macrophages (Figure 2), and cerebral vasculitis was associated with surrounding malacia. Pulmonary alveoli were flooded with lightly eosinophilic fluid (edema) containing scattered erythrocytes, plasma cells, and macrophages. Hepatocytes were diffusely expanded, and floccular vacuolation was compressing adjacent sinusoids. Small amounts of viral antigen were detected in a necrotic glomerulus and within the media of a renal arteriole by immunoperoxidase staining.

**Table 1:** Gross and histopathologic findings in tissues of Hendra virus–infected dog, Australia, 2013*

Sample	Gross pathology	Histopathology
Pharynx	ND	NSF
Soft palate	ND	NSF
Tonsil	Moderate	NA
Lymph node		
Submandibular	ND	NSF
Mandibular	Mild	NSF
Bronchial	Moderate	Moderate
Tracheobronchial	Moderate	Moderate
Axillary	ND	NSF
Inguinal	ND	NSF
Lung	Moderate	Mild
Myocardium	Mild	Mild
Spleen	Mild	Mild
Liver	Mild	Mild
Kidney	Moderate	Marked
Adrenal gland	ND	NSF
Bladder	ND	NSF
Intestine		
Small	ND	NSF
Large	ND	Mild
Brain		
Olfactory	ND	Moderate
Occipital	ND	Moderate
Cerebellum	ND	Moderate
Brain stem	ND	Moderate
Meninges	ND	Moderate
Spinal cord	ND	NA
Turbinate	ND	NSF
Trigeminal ganglion	ND	NA
Brachial nerve	ND	NA

*NA, tissue not available; ND, not detected; NSF, no significant changes found.

An extensive range of fresh tissues and swab samples were collected for testing by qRT-PCR, and HeV RNA was found in many of the tissues (Table 2). No virus was isolated from any of the tissues in cell culture. Serum collected at the time of euthanasia was positive by ELISA; virus neutralization titer was 128. All other animals on the farm remained seronegative when sampled 4 weeks after the infected dog had been euthanized. Laboratory methods are described in the Technical Appendix.

**Table 2:** Hendra virus RNA levels in tissues and blood of Hendra virus–infected dog, Australia, 2013*

Sample	RNA level†
Blood (in EDTA)	33.36
Pharynx	36.52
Soft palate	35.08
Tonsil	36.12
Lymph node	
Submandibular	ND
Mandibular	33.91
Bronchial	28.32
Tracheobronchial	28.26
Axillary	32.87
Inguinal	33.91
Spleen	29.64
Lung	35.00
Myocardium	28.62
Liver	27.65
Kidney	29.03
Adrenal	34.06
Bladder	33.68
Intestine	
Small	35.01
Large	ND
Spinal cord	28.67
Brain	
Olfactory	34.84
Occipital	34.38
Cerebellum	ND
Brain stem	ND
Meninges	ND
Turbinate	ND
Trigeminal ganglion	ND
Brachial nerve	ND

*ND, not detected.  †Cycle threshold.

## Conclusions

Dogs and cats have been infected with HeV under experimental conditions. Previously, a dog located on the same property as 3 infected horses in Queensland, Australia, was found to be seropositive (*6*) without having shown clinical signs. The dog reported in this article, which also remained clinically healthy, was naturally infected and was identified during the acute stages of infection. Viral RNA was detected in this animal 12 days after euthanasia of the clinically affected horse. The dog was known to have been in close contact with the live infected horse and is suspected of having been exposed to its blood after the horse was euthanized. The epidemiologic and laboratory evidence supports transmission of HeV from horse to dog. In horses naturally infected with HeV, the development of neutralizing antibodies is associated with virus clearance from the infected animal. The detection of seroconversion and rising neutralizing antibody titers in canine serum collected ≈14 and then 16 days after putative virus exposure is consistent with the early stages of HeV infection and aligns with the low viral RNA levels in blood and a wide range of tissues (the highest levels were found in liver, bronchial lymph node, kidney, and myocardium). Failure to isolate virus in cell culture was probably the result of increasing antibody levels. It is difficult to establish from the qRT-PCR results whether virus replication occurred in tissues such as kidney, liver, myocardium, and spinal cord or whether this finding represents residual RNA from blood. However, the levels in these sites were 10–100-fold higher than that in blood, suggesting either local replication or accumulation of viral RNA. Very low levels of viral RNA were detected in the soft palate, pharynx, and tonsil, although virus was not detected in nasal, oral, rectal, or vaginal swab samples and urine. The risk for transmission of HeV from infected dogs to other susceptible species—including humans—remains unknown.

The histopathologic finding of widespread necrotizing vasculitis supports the current understanding of the pathogenesis of HeV infection, during which virus binds to the endothelial ephrin-B2 transmembrane protein receptor (*7*) and localizes in vessel walls, leading to endothelial cell damage. The most severe vascular lesions were found in kidney, brain, and lymph nodes; the lungs were relatively spared, and fulminant pulmonary edema and interstitial pneumonia were not significant findings in this case.

The route of infection for the dog reported here is unknown, but the dog was in close contact with the infected horse and is suspected to have had contact with its blood. Because viral loads in acutely infected horses are usually very high, dogs can be readily infected and should be kept away from infected horses, which seem to be efficient amplifying hosts.

Technical Appendix Additional laboratory methods used to detect Hendra virus in dog, Australia, 2013.
