# Supplementary material for: Hendra Virus Infection in Dog, Australia, 2013
Source: Emerg Infect Dis. 2015 Dec;21(12):2182–5. doi: 10.3201/eid2112.151324 (PMC4672422; doi:10.3201/eid2112.151324)
Supplement: Technical Appendix — Additional laboratory methods used to detect Hendra virus in dog, Australia, 2013. [file 15-1324-Techapp-s1.pdf]

Article DOI: http://dx.doi.org/10.3201/eid2112.151324

Hendra Virus Infection in Dog, Australia, 2013

Technical Appendix

Laboratory Methods

Serology and Virology

Antibodies to Hendra virus were detected in an ELISA (*1*) using peroxidase conjugated anti-equine IgG for horse serum and a recombinant protein A/G conjugate for other species or by virus neutralizsation test (*1*). Virus isolation was attempted by culture of blood or tissue samples on Vero cells (*1*).

Histopathology

Tissues were collected and fixed in 10% neutral buffered saline before routine histological processing and staining with hematoxylin and eosin using standard techniques.

Immunohistochemistry

Immunoperoxidase staining was carried out using a rabbit polyclonal antibody raised against the Nipah virus N protein (*2*)

Detection of viral RNA

RNA encoding the M gene of HeV was detected by the use of a real time reverse transcription polymerase chain reaction (qRT-PCR) assay (*3*). To avoid the generation of aerosols during the extraction of RNA from fresh tissue samples, small fragments (≈15–20mg) of tissue were digested enzymatically (*4*). Total nucleic acid was purified from 25uL of whole unclotted blood and the supernatant from tissue digests or 50uL of viral transport medium from swabs and serum using an RNA extraction kit (MagMax 96 viral RNA, Ambion, Austin, Texas) on a magnetic particle handling system (Kingfisher, Thermo, Finland) according to the manufacturer’s instructions. Five microliters of purified nucleic acid was added to 20uL of AgPath (Ambion, Austin, Texas) mastermix and run on a thermocycler (ABI 7500, Applied Biosystems, Foster City, California) for 45 cycles under standard cycling conditions. Results were expressed as cycle-threshold units.

References

1. Daniels P, Ksiazek T, Eaton BT. Laboratory diagnosis of Nipah and Hendra virus infections. Microbes Infect. 2001;3:289–95. PubMed http://dx.doi.org/10.1016/S1286-4579(01)01382-X

2. Middleton DJ, Westbury HA, Morrissy CJ, van der Heide BM, Russell GM, Braun MA, et al. Experimental Nipah virus infection in pigs and cats. J Comp Pathol. 2002;126:124–36. PubMed http://dx.doi.org/10.1053/jcpa.2001.0532

3. Smith IL, Halpin K, Warrilow D, Smith GA. Development of a fluorogenic RT-PCR assay (TaqMan) for the detection of Hendra virus. J Virol Methods. 2001;98:33–40. PubMed http://dx.doi.org/10.1016/S0166-0934(01)00354-8

4. Jenkins C, Hick P, Gabor M, Spiers Z, Fell SA, Gu X, et al. Identification and characterisation of an ostreid herpesvirus-1 microvariant (OsHV-1 micro-var) in *Crassostrea gigas* (Pacific oysters) in Australia. Dis Aquat Organ. 2013;105:109–26. PubMed http://dx.doi.org/10.3354/dao02623
